# Prosperity and Cancer

**Published:** 1897-12-18

**Authors:** 


### Prosperity and Cancer.

It does not do to be always looking on the rosy side of things, and although we may well congratulate ourselves on the steady lowering of the death-rate which has taken place within the last fifty years, it is impossible to regard entirely without disquietude the simultaneous increase which has occurred in the registered deaths from cancer.

The figures certainly are startling. In 1840, cancer caused 2,786 deaths in this country, being 1 in 129 of the total mortality, or 177 per million living. In 1894 it caused 21,422 deaths, the proportion being 1 in 23 of the total mortality, or 713 per million living. Thus, in a little over fifty years, the deaths from cancer in proportion to the population had increased more than four-fold, while in proportion to the total deaths from all causes they had increased more than five-fold in the same time.

This increase of cancer cannot, we think, be explained away (although we may grant that improved registration may have had something to do with the transference of a certain number of cases from the category of phthisis to that of cancer), and what is curious is the fact, to which attention has been lately drawn, that in other countries as well as in our own the growth of wealth and the growth of cancer have seemed to run together. For long cancer has been looked upon as a poor man's disease. It has, in fact, come to be spoken of as "morbus miseriæ," and a recent author has even said, "My experience is that uterine cancer occurs only among the working classes." Mr. Roger Williams, however, writing in the *Edinburgh Medical Journal*, directly traverses this opinion, and some of the statements which he makes are sufficiently striking. He points to the enormous growth of material wealth which has taken place during the years that have seen this increase of the death-rate from cancer, and asserts that "the Registrar-General's reports show that the cancer mortality is *lowest* where the conditions of life are hardest, the surroundings the most squalid, the density of the population greatest, the tubercle mortality highest, the general mortality highest, and where sanitation is least perfect—in short, among the industrial classes in our great towns; whereas amongst the wealthy and well-to-do—where life is easiest—and among the agricultural community, there the cancer mortality is highest."

He goes on to say that in London, where the wealth of the nation is "clotted," there the cancer mortality is at its maximum, and that this mortality is highest of all in those parts of the metropolis where the well-to-do most abound, that of west London being double that of the east.

We cannot, however, but feel that, when it is stated that a disease so common among the agricultural community is caused by "high feeding and easy living," there is a weak spot in the argument; and this very anomaly forces upon us, what other reasons would suggest, that, correct as may be the figures and statements made in regard to the coexistence of an increase of cancer with a growth of material wealth, the explanation is something very different from anything like ease and high feeding. We might, in fact, be tempted to ask whether the wide generalisation propounded by the late Henry George as to the constant relation between progress and poverty might not explain the prevalence of cancer in progressive countries, even on the older hypothesis that cancer is truly a "morbus miseriæ." Putting that on one side, however, we may fairly ask whether the connection between cancer and prosperity is not purely accidental, and whether the true relationship is not one between cancer and such conditions of existence as enable people to escape other causes of death.

If we take the death-rate from phthisis as showing an index of the misery and insanitary surroundings of any group of persons, it is no doubt a striking thing that the cancer death-rate should vary in an inverse ratio. But if we recognise that, where there is such misery, the people die young, we seem to see how it happens that cancer, which is largely a disease of advancing life, prevails most in districts where people live on into the cancer age; and this may well be the explanation of the prevalence of the disease among the agricultural population, who notoriously live long.

We doubt, however, whether the increase of cancer during recent years can be explained by such simple means. Much is now being done in the investigation of this disease. A constant hunt is being made for some underlying microbic condition; the mode in which cancer spreads is being most carefully investigated both clinically and in the laboratory; the influences which tend to make it grow, and those under which—in some cases—it seems to shrivel and lose its virulence, are all being carefully observed; and we feel sure that it is in these directions that help must be sought rather than from mere statistics, or from attempts to connect the disease with either poverty or progress.

